# Patients with scans without evidence of dopaminergic deficit (SWEDD) do not have early Parkinson’s disease: Analysis of the PPMI data

**DOI:** 10.1371/journal.pone.0246881

**Published:** 2021-02-10

**Authors:** Jeong Won Lee, Yoo Sung Song, Hyeyun Kim, Bon D. Ku, Won Woo Lee

**Affiliations:** 1 Department of Nuclear Medicine, Catholic Kwandong University College of Medicine, International St. Mary’s Hospital, Incheon, Republic of Korea; 2 Department of Nuclear Medicine, Seoul National University Bundang Hospital, Seongnam, Republic of Korea; 3 Department of Neurology, Catholic Kwandong University College of Medicine, International St. Mary’s Hospital, Incheon, Republic of Korea; 4 Institute of Radiation Medicine, Medical Research Center, Seoul National University, Seoul, Republic of Korea; Maastricht University, NETHERLANDS

## Abstract

**Background:**

To evaluate whether patients with scans without evidence of dopaminergic deficit (SWEDD) have early Parkinson’s disease (PD).

**Methods:**

The clinical characteristics, striatal specific binding ratios (SBRs), and the indices of I-123 FP-CIT SPECT images of 50 SWEDD patients, 304 PD patients, and 141 healthy controls were acquired from the Parkinson’s Progression Markers Initiative (PPMI) data and evaluated during a 2-year clinical follow-up period.

**Results:**

Of the 50 subjects with SWEDD, PD was confirmed in 13 subjects (the PD-SWEDD group), while the remaining 37 subjects had other diseases (the Other-SWEDD group). Striatal SBR values and striatal asymmetry indices of the PD group were significantly different with those of the PD-SWEDD and Other-SWEDD groups at both baseline and after 2 years (*p* < 0.001). Putaminal SBR values of the PD-SWEDD group were significantly decreased after 2 years (*p* < 0.05). There was no difference of the SBR values between baseline and after 2 years in the Other-SWEDD group. A baseline MDS-UPDRS III score matched comparison of the PD and PD-SWEDD group was done due to the large difference of the subject numbers. Striatal SBR values and striatal asymmetry indices were significantly different (*p* < 0.001) between the two groups at both baseline and after 2 years, but there were no significant difference with respect to the MDS-UPDRS III scores after 2 years between the two groups.

**Conclusion:**

The different SBR values and asymmetry indices between the PD and PD-SWEDD groups at baseline and after 2 years indicate that SWEDD may not be early PD, but rather a different disease entity.

## Introduction

Scans without evidence of dopaminergic deficit (SWEDD) refers to patients clinically diagnosed with Parkinson’s disease (PD), but show normal findings on presynaptic dopaminergic imaging [1]. According to previous clinical trials, the proportion of these SWEDD patients in the entire PD population could be as high as 20% [1, 2]. While SWEDD refers primarily to the presynaptic dopaminergic image findings and not to the etiology, its diagnosis and clinical features remain controversial. There is evidence that SWEDD may simply be a misdiagnosis of other different conditions, including essential tremor, dystonic tremor, or other neurodegenerative disorders [3–5]. It has been proposed that the clinical features of SWEDD differ from PD in that subjects may present with intact olfactory function [6], different frequency of non-motor symptoms [7, 8], non-responsiveness to levodopa treatment [9], and minimal progression of dopaminergic denervation during the follow-up period [3]. In contrast, some studies suggest that SWEDD patients truly have dopaminergic degeneration despite normal imaging results, simply because they are diagnosed in the early stages of PD. Significant portion of patients evaluated as SWEDD at baseline were confirmed with PD after a certain period of time during the follow-up period, showing a progression of presynaptic dopaminergic degeneration [2, 5, 10].

The primary goal of this study was to evaluate whether SWEDD actually falls into the category of early PD, or whether it is a misdiagnosis of other diseases. In the present study, we evaluated the clinical symptoms, dopaminergic denervation, and indices measured with [I-123] N-ω-fluoropropyl- 2β-carbomethoxy- 3β-(4-iodophenyl) nortropane (I-123 FP-CIT) single photon emission computed tomography (SPECT) images in SWEDD patients, PD patients, and healthy controls during a 2-year clinical follow-up period, from the Parkinson’s Progression Markers Initiative (PPMI) data. Moreover, we performed a symptom-severity-matched group analysis between SWEDD and PD patients to evaluate the rate of dopaminergic denervation of same clinical stages.

## Methods

### Patients

Data of healthy controls, PD patients, and SWEDD patients were acquired from the online PPMI database (http://www.ppmi-info.org) in July 2019. The inclusion criteria adopted for this study was outlined in the PPMI database. In short, the inclusion criteria for the PD group were as follows: patients aged 30 years or more, irrespective of gender; scale of I or II on the Hoehn and Yahr (H&Y) scale at baseline; confirmation via presynaptic dopaminergic scans; and no expectation of PD medication within six months from baseline. Patients on PD-related medication were excluded. The inclusion criteria for the SWEDD group were the same as those for the PD group, except that subjects required to have no evidence of dopaminergic deficit in presynaptic dopaminergic scans. The visual interpretation for normality was determined by the imaging core of PPMI, and was informed to the referring physician. The inclusion criteria for the healthy control (HC) group were as follows: subjects aged 30 years or more at the time of screening, irrespective of gender; showing no signs of active significant neurological disorders; have no first-degree relatives with PD; and have no history of dopamine transporter-interfering drug use. The clinical diagnosis of PD and SWEDD subjects were reassessed using the Clinical Diagnosis and Management Questionnaire at the 24-month visit; this served as the final clinical diagnosis. Data of PPMI were acquired in accordance with the relevant guidelines and regulations, and written informed consent were obtained from all participants before obtaining relevant clinical data. In accordance with the 1964 Declaration of Helsinki and its later amendments, written informed consent was collected from all participating subjects. This multicenter study was approved by all respective local Institutional review boards (IRBs, 33 institutions, listed at https://www.ppmi-info.org/about-ppmi/ppmi-clinical-sites).

Subjects without available baseline I-123 FP-CIT SPECT scans in the PPMI database were excluded, resulting in a total of 50 SWEDD patients (age 60.8 ± 10.1 years, male: female = 30: 20), 304 PD patients (age 61.0 ± 9.6 years, male: female = 199: 105), and 141 HCs (age 60.7 ± 11.1 years, male: female = 88: 53). Participants’ clinical data, including age, gender, subjects’ weight, previous symptom duration period at enrollment, H&Y scales, Scales for outcomes in Parkinson’s disease-Autonomic dysfunction (SCOPA-AUT) scores, Movement disorder society-Unified Parkinson’s disease rating scale (MDS-UPDRS) II/III scores, and I-123 FP-CIT SPECT analysis results were collected from the PPMI data. The clinical data at the baseline and at 2-year of follow-up were evaluated for the SWEDD and PD groups. For subtyping SWEDD and PD into tremor dominant (TD), postural instability/gait difficulty (PIGD), indeterminant subtypes, and UPDRS items were used to calculate the mean TD and PIGD scores [11]. Subjects were classified as TD if the ratio between the mean tremor score and the mean PIGD score was ≧ 1.15, as PIGD if ≦ 0.90, and as indeterminant if between 0.90 and 1.15.

### I-123 FP-CIT SPECT analysis

Upon enrollment and at 2-year follow-up, SPECT scans were performed 4 ± 0.5 hours after I-123 FP-CIT injection (111–185 MBq, 3–5 mCi). At the 2-year follow-up, scans were acquired in 47 SWEDD patients and 265 PD patients. Images were reconstructed iteratively, with no filtering. To maintain uniform data quality among multiple institutions, the core imaging lab of PPMI visited each respective center for technical setup. A dedicated software (PMOD, PMOD Technologies, Zurich, Switzerland) was used for analysis. Specific binding ratios (SBRs, (uptake of target region/uptake of occipital cortex)-1) of the right and left caudate nucleus and putamen were acquired. For the HC group, minimum SBR values among the bilateral caudate nucleus and putamen were selected for analysis [12]. For the SWEDD and PD groups, caudate nucleus and putamen regions related to the dominant side of symptoms based on the PPMI database were selected for analysis [13]. The asymmetry index was calculated as the difference between the left and right SBR values divided by the mean SBR value. Percent decrease (1- (SBR at 2 years/SBR at baseline)) of striatal SBRs after 2 years were calculated (SBR % dec) in the SWEDD and PD groups.

### Statistical analysis

Medcalc version 19.2 (MedCalc Software Ltd, Belgium) was used for analysis. Striatal SBRs and demographic factors were compared among healthy controls, SWEDD, and PD groups using one-way ANOVA with Scheffé test for post-hoc, or Kruskal-Wallis test with Conover test for post-hoc. SBR related values and demographic factors were compared between the baseline and 2-year follow-up periods of respective groups, and between the MDS-UPDRS III matched PD and PD-SWEDD group using t-test, or Mann-Whitney test, or paired sample t-test. Chi-square test was performed to assess any differences in subtypes between the SWEDD and PD groups. Statistical significance was set at *p <* 0.05. Case-control matching scores was applied for the PD and PD-SWEDD groups, by matching subjects with equal baseline MDS-UPDRS III scores.

## Results

### Demographic characteristics

Fifty SWEDD subjects were reassessed at the 2-year follow-up. A clinical diagnosis of PD was confirmed in 13 patients (the PD-SWEDD group), and other diseases were confirmed in 37 patients (the Other-SWEDD group). The Other-SWEDD group included 1 patient with Alzheimer’s disease, 9 with essential tremor, 5 with psychogenic illness, 1 with vascular parkinsonism, 1 with spinocerebellar ataxia, 9 with other neurologic diseases, and 11 with no PD nor other neurological disorders. The baseline characteristics of the respective groups are presented in Table 1. There were no significant differences of age at enrollment, body weight, and gender between the groups. Moreover, there were no significant differences of symptom durations, and age of symptom onsets between the PD and SWEDD groups. H&Y scales in the PD group was significantly higher than those in the Other SWEDD group (*p* < 0.001), but not higher than those in the PD-SWEDD group. The SCOPA-AUT scores were significantly lower in the HC group when compared with the two other groups; and these scores were lower in the PD group than the Other-SWEDD group (*p* < 0.001) at baseline. The differences of SCOPA-AUT scores between the PD group and the Other-SWEDD group disappeared after 2 years of follow-up. The MDS-UPDRS II scores were lower in the HC group than in the other groups at baseline (*p* < 0.001). There were no significant differences of the MDS-UPDRS II scores between the PD group and SWEDD groups at the 2-year follow-up. The MDS-UPDRS III scores were lower in the HC group than in the other groups, and these scores were lower in the Other-SWEDD group than in the PD and PD-SWEDD groups (*p* < 0.001). The MDS-UPDRS III scores of the PD, PD-SWEDD, and Other-SWEDD groups were different from one another after 2 years of follow-up (*p* < 0.001). There was no significant difference (*p* = 0.45) with respect to the distribution of subtypes between the PD group and the SWEDD groups at baseline (Table 2).

**Table 1:** Clinical characteristics of the HC, PD, and SWEDD groups.

	HC (141)	PD (304)	PD-SWEDD (13)	Other-SWEDD (37)	*p*-value
Age at enroll	60.7 ± 11.1	61.0 ± 9.6	59.8 ± 10.8	61.1 ± 9.9	0.97
Gender (M: F)	88: 53	199: 105	7: 6	23: 14	0.78
Weight (kg)	78.5 ± 15.8	81.3 ± 17.1	85.5 ± 11.2	85.5 ± 18.7	0.08
Symptom duration (months)		23.1 ± 22.3	13.8 ± 8.8	28.1 ± 33.8	0.20
Age of symptom onset		59.0 ± 9.8	58.0 ± 11.3	58.6 ± 10.5	0.92
H&Y scales		1.55 ± 0.51^d^	1.50 ± 0.67	1.18 ± 1.00^b^	< 0.001
SCOPA-AUT, baseline	6.4 ± 5.0^b^^,^^c^^,^^d^	9.5 ± 6.8^a^^,^^d^	14.9 ± 9.8^a^	12.7 ± 8.4^a^^,^^b^	< 0.001
SCOPA-AUT, 2yrs		11.4 ± 7.1	14.8 ± 9.3	12.5 ± 7.3	0.20
MDS-UPDRS II, baseline	1.5 ± 1.1^b^^,^^c^^,^^d^	6.6 ± 4.1^a^	5.3 ± 3.3^a^	6.9 ± 4.6^a^	< 0.001
MDS-UPDRS II, 2yrs		8.7 ± 5.0	10.0 ± 7.9	9.1 ± 9.2	0.37
MDS-UPDRS III, baseline	1.2 ± 2.1^b^^,^^c^^,^^d^	20.3 ± 8.6^a^^,^^d^	17.8 ± 9.1^a^^,^^d^	12.8 ± 9.0^a^^,^^b^^,^^c^	< 0.001
MDS-UPDRS III, 2yrs		26.3 ± 11.1^c^^,^^d^	20.2 ± 16.1^b^^,^^d^	12.4 ± 13.0^b^^,^^c^	< 0.001

For a particular variable, values in superscripts indicate groups of statistically significant difference on post-hoc. Values represent:

a for HC,

b for PD,

c for PD-SWEDD, and

d for Other-SWEDD groups. Values are mean ± SD.

**Table 2:** Subtypes of the PD and the SWEDD groups.

	PD	PD-SWEDD	Other-SWEDD
Tremor dominant	245	13	29
Indeterminant	16	0	3
PIGD	43	0	5

### SBRs and related values of HC, PD, and SWEDD groups, at baseline and 2 years of follow-up

The 2-year follow-up scans were acquired in 265 PD patients and 47 SWEDD patients (87.1% and 94.0% of baseline patients, respectively). There were no significant differences of any clinical factors listed on Table 1, between the PD patients that had the 2-year follow-up scans and the PD patients that had not. The baseline caudate nucleus and putamen values of the PD group were significantly lower than those of the HC and SWEDD groups (*p* < 0.001); however, there was no significant difference between the HC and the SWEDD groups. The caudate nucleus and putamen SBR values of the PD group were significantly lower than those of the SWEDD groups at the 2-year follow-up (*p* < 0.001). The asymmetry index was higher in the PD group than in the other groups, both at baseline and at 2-year follow-up (*p* < 0.001). The SBR % dec of the PD group was significantly higher than that of the Other-SWEDD group after 2 years of follow-up, in both caudate nucleus (*p* < 0.001) and putamen (*p* < 0.05) (Table 3).

**Table 3:** Comparison of SBRs and related values among the HC, PD, and SWEDD groups.

	HC	PD	PD-SWEDD	Other-SWEDD	*p*-value
SBR					
Caudate nucleus, baseline	3.0 ± 0.6^b^	2.0 ± 0.6^a^^,^^c^^,^^d^	2.9 ± 0.6^b^	2.9 ± 0.6^b^	< 0.001
Putamen, baseline	2.2 ± 0.5^b^	0.8 ± 0.3^a^^,^^c^^,^^d^	2.0 ± 0.7^b^	2.1 ± 0.5^b^	< 0.001
Caudate nucleus, 2 yrs		1.7 ± 0.5^c^^,^^d^	2.8 ± 1.0^b^	2.7 ± 4.4^b^	< 0.001
Putamen, 2 yrs		0.6 ± 0.3^c^^,^^d^	1.7 ± 0.7^b^	2.0 ± 0.5^b^	< 0.001
Asymmetry index					
Caudate nucleus, baseline	7.7 ± 5.5^b^	19.0 ± 13.3^a^^,^^c^^,^^d^	8.9 ± 6.5^b^	7.6 ± 4.6^b^	< 0.001
Putamen, baseline	10.8 ± 8.7^b^^,^^c^	37.1 ± 24.6^a^^,^^c^^,^^d^	20.0 ± 13.7^a^^,^^b^	11.8 ± 10.2^b^	< 0.001
Caudate nucleus, 2 yrs		19.8 ± 15.2^c^^,^^d^	12.8 ± 15.2^b^	7.4 ± 4.8^b^	< 0.001
Putamen, 2 yrs		33.0 ± 22.6^c^^,^^d^	22.5 ± 19.4^b^^,^^d^	10.3 ± 8.0^b^^,^^c^	< 0.001
2 yr SBR % dec					
Caudate nucleus		14.9 ± 18.0^d^	4.9 ± 19.2	2.7 ± 14.9^b^	< 0.001
Putamen		15.1 ± 26.2^d^	15.9 ± 23.5	1.2 ± 22.6^b^	< 0.05

For a particular variable, values in superscripts indicate groups of statistically significant difference on post-hoc. Values represent:

a for HC,

b for PD,

c for PD-SWEDD, and

d for Other-SWEDD groups. Values are mean ± SD.

When comparing the caudate nucleus and putamen SBR values between the baseline and after 2 years of follow-up, there was a significant decrease of caudate nucleus SBR, putamen SBR, and the putamen asymmetry index in the PD group (p < 0.001, < 0.001, and < 0.01, respectively). However, in the PD-SWEDD group, only the putamen SBRs decreased significantly after 2 years of follow-up (*p* < 0.05). There was no significant change in any of the indices of the Other-SWEDD group (Table 4).

**Table 4:** Comparison of SBR values between baseline and 2 years of follow-up.

	Baseline	2 yrs	*p*-value
PD	n = 304	n = 265	
Caudate nucleus, SBR	2.0 ± 0.6	1.7 ± 0.5	< 0.001
Putamen, SBR	0.8 ± 0.3	0.6 ± 0.3	< 0.001
Caudate nucleus, asymmetry index	19.0 ± 13.6	19.8 ± 15.2	0.35
Putamen, asymmetry index	37.4 ± 23.9	33.0 ± 22.6	< 0.01
PD-SWEDD	n = 13	n = 13	
Caudate nucleus, SBR	2.9 ± 0.7	2.8 ± 1.0	0.61
Putamen, SBR	2.0 ± 0.7	1.7 ± 0.7	< 0.05
Caudate nucleus, asymmetry index	9.4 ± 6.4	12.8 ± 15.2	0.42
Putamen, asymmetry index	20.8 ± 14.0	22.5 ± 19.4	0.75
Other SWEDD	n = 37	n = 34	
Caudate nucleus, SBR	2.8 ± 0.6	2.7 ± 0.4	0.11
Putamen, SBR	2.0 ± 0.5	2.0 ± 0.5	0.29
Caudate nucleus, asymmetry index	7.3 ± 4.6	7.4 ± 4.8	0.98
Putamen, asymmetry index	11.9 ± 10.2	10.3 ± 8.0	0.41

Values are mean ± SD.

### Comparison of MDS-UPDRS III score matched groups

**Fig 1:**
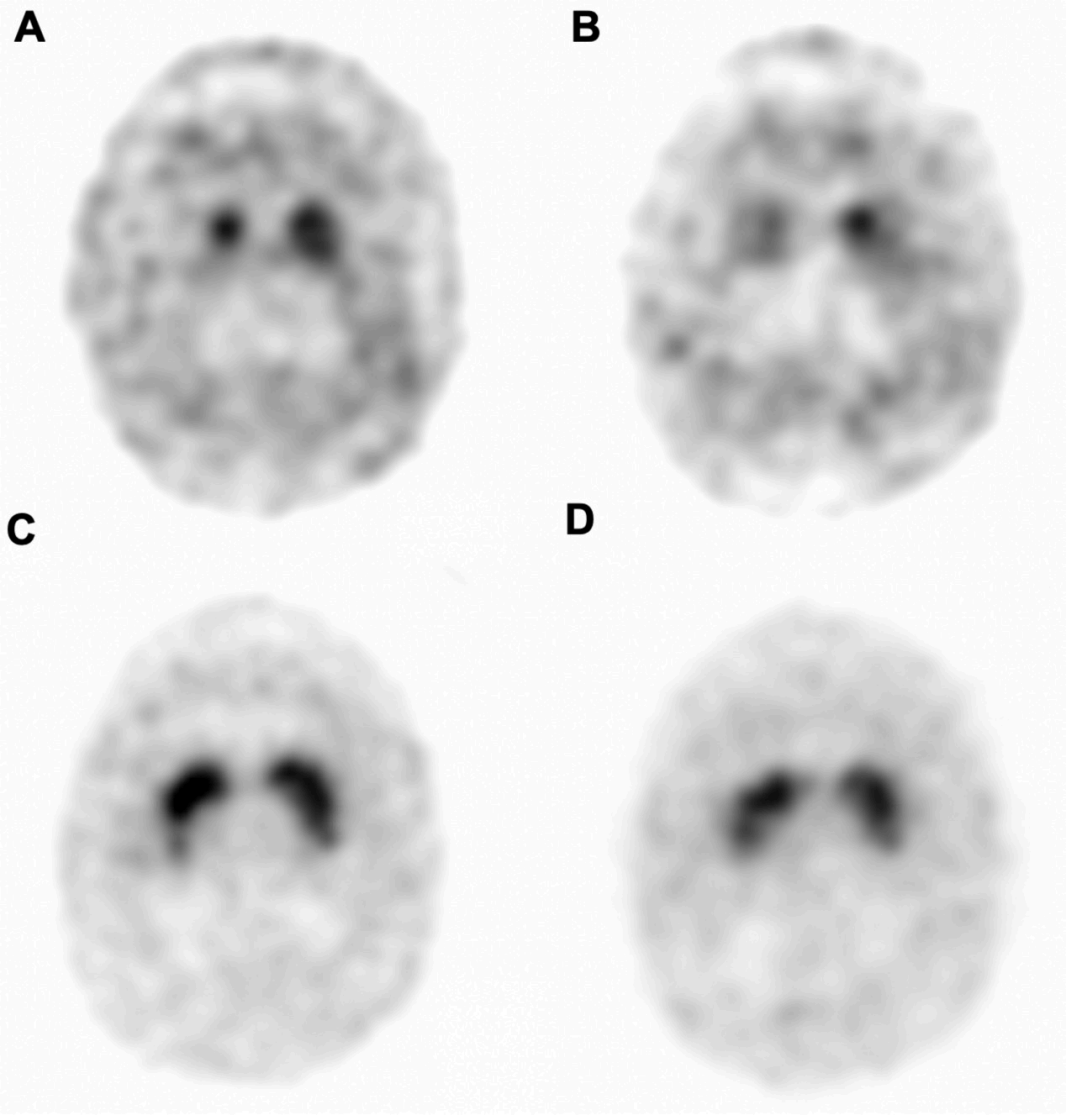
Representative cases of a PD and PD-SWEDD patients. I-123 FP-CIT SPECT images of a PD (A, B) and PD-SWEDD (C, D) patients at baseline (A, C) and at 2-year follow-up (B, D). Baseline MDS-UPDRS III scores were 36 for both patients. 2-year MDS-UPDRS III scores were 59 for the PD patient, and 61 for the PD-SWEDD patient.

Case-control matching of the baseline MDS-UPDRS III scores was applied to the PD and PD-SWEDD groups, drawing 13 patients from each group. 2-year follow-up scans were performed in 10 patients for the PD group, and SBR related indices were analyzed with these 10 matched subjects. There were no significant differences of age at enroll, symptom duration, age of symptom onset, and gender between the two matched groups. Body weight of the PD-SWEDD group was significantly higher than that of the PD group (*p* < 0.01). The caudate nucleus and putamen SBRs of the PD group was significantly lower than those of the SWEDD group, at both baseline and at 2-year follow-up (p < 0.01, < 0.001, respectively). The asymmetry indices of the caudate nucleus and putamen were both higher in the PD group than in the PD-SWEDD group, at both baseline (*p* < 0.05, < 0.01) and at 2-year follow-up (*p* < 0.01, < 0.05). There was no significant difference with respect to the SBR % dec of the caudate nucleus and putamen between the PD and PD-SWEDD groups. There was no significant difference with respect to the SCOPA-AUT scores at baseline; however, at 2-year follow-up, the SCOPA-AUT scores were higher in the PD-SWEDD group (*p* < 0.05.) There was no significant difference with respect to the MDS-UPDRS II scores at both baseline and at 2-year follow-up. Moreover, there was also no significant difference with respect to the MDS-UPDRS III scores at the 2-year follow-up (Table 5). Representative cases are illustrated in Fig 1.

**Table 5:** MDS-UPDRS III matched comparison of the PD and PD-SWEDD group.

	PD (n = 13)	PD-SWEDD (n = 13)	*p-*value
Age at enroll	59.2 ± 7.2	59.8 ± 10.8	0.89
Symptom duration (months)	18.5 ± 9.1	13.8 ± 8.8	0.19
Age of symptom onset	57.6 ± 6.8	58.0 ± 11.3	0.93
Gender (M: F)	5: 8	7: 6	0.44
Weight (kg)	70.1 ± 11.7	85.5 ± 11.3	<0.01
Caudate nucleus SBR, baseline	1.9 ± 0.5	2.9 ± 0.6	< 0.01
Putamen SBR, baseline	0.8 ± 0.3	2.0 ± 0.7	< 0.001
Caudate nucleus SBR, 2 yrs	1.6 ± 0.4	3.1 ± 1.0	< 0.01
Putamen SBR, 2 yrs	0.6 ± 0.2	1.9 ± 0.6	< 0.001
Caudate nucleus, asymmetry index, baseline	20.3 ± 14.4	8.9 ± 6.5	< 0.05
Putamen asymmetry index, baseline	52.6 ± 36.0	20.0 ± 13.7	< 0.01
Caudate nucleus asymmetry index, 2 yrs	28.2 ± 11.7	9.0 ± 7.6	< 0.01
Putamen asymmetry index, 2 yrs	38.2 ± 26.5	17.4 ± 7.6	< 0.05
Caudate nucleus SBR, % dec	10.1 ± 14.6	0.0 ± 18.0	0.19
Putamen SBR, % dec	19.3 ± 19.5	11.6 ± 24.1	0.43
SCOPA-AUT, baseline	8.5 ± 6.6	14.9 ± 9.8	0.05
SCOPA-AUT, 2yrs	8.7 ± 2.9	13.9 ± 4.7	< 0.05
MDS-UPDRS II, baseline	6.4 ± 5.1	5.3 ± 3.3	0.36
MDS-UPDRS II, 2yrs	8.6 ± 5.6	10.1 ± 9.0	0.49
MDS-UPDRS III, baseline	17.8 ± 9.1	17.8 ± 9.1	1.00
MDS-UPDRS III, 2yrs	25.8 ± 15.3	22.0 ± 18.8	0.48

Values are mean ± SD.

## Discussion

To the best of our knowledge, there seem to be several limitations in previous studies evaluating the serial changes of dopaminergic denervation in SWEDD patients. Presynaptic dopamine images were interpreted visually, and not quantitatively, which raises the possibility of interobserver variability and inconsistent diagnosis [2]. Furthermore, the dopaminergic denervation rate of SWEDD subjects was not compared with that of symptom severity- or disease duration-matched PD subjects, making it difficult to discern whether SWEDD subjects are early PD [3, 10].

In our study, there were no significant baseline differences of the MDS-UPDR III scores between the PD group and the PD-SWEDD group, while the MDS-UPDR III scores of the PD group were significantly higher than those of the Other-SWEDD group. After 2 years of follow-up, the MDS-UPDRS III scores of the PD group was significantly higher than those of the PD-SWEDD and the Other-SWEDD groups. This implies that the PD group has a more aggressive course of progression, considering that there are no significant differences in the age of symptom onset and symptom durations between the PD and SWEDD groups. However, the 2-year percent decrease was not significantly different between the PD group and the PD-SWEDD group, while all other SBR related indices were worse in the PD group compared with the SWEDD groups. This may be due to the exponential decrease pattern of dopaminergic transporter density in PD, where the progression rate in the early phase of PD is higher in the earlier phases [14]. Therefore, the percent decrease in the PD group is low since they are in the later phases of the disease. However, the PD-SWEDD group also has a low percent decrease, which is comparable to the previously reported dopaminergic denervation rate due to normal aging in healthy controls [12, 15].

The asymmetry index is also a good predictor for a correct diagnosis, since the unilateral appearance of clinical symptoms is a hallmark feature of PD [16, 17]. Moreover, PD patients with a higher asymmetry index also have a higher response rate to the levodopa treatment, which is also an important indicator for the diagnosis of PD [18]. In our study, the asymmetry indices of the PD group were significantly higher than any other groups at both the baseline and at 2-year follow-up, which is a relevant characteristic. However, the PD-SWEDD group showed significantly higher putaminal asymmetry indices compared with the HC group at baseline and with the Other-SWEDD group after 2 years of follow-up. This indicates that the PD-SWEDD group may have a different etiology from the PD and Other SWEDD groups.

Finally, we evaluated the MDS-UPDRS III score-matched features of the PD and the PD-SWEDD groups. Matching was done to balance the number of subjects within each group, in order to reduce the variance in the parameters of interest, with expectations of improved statistical efficiency [19, 20]. Our study indicated that there were no significant differences of the MDS-UPDRS III score after 2 years of follow-up, while there was no significant difference in the age of onset and symptom duration. However, the SBRs and related indices were more aggravated in the PD group, except for the percent decrease. Among patients with the same degree of motor symptoms, PD patients had significantly more progressed dopaminergic denervation than PD-SWEDD patients, while there was no significant difference in the MDS-UPDRS III scores after 2 years. However, the SCOPA-AUT scores were higher in the PD-SWEDD group than in the PD group. A previous study reported that autonomic dysfunction is present in most SWEDD patients; however, to the best of our knowledge, there is no correlation between the degree of autonomic dysfunction and the degree of Parkinsonism [21]. Our study indicates that PD-SWEDD patients, when compared with PD patients, have a similar degree of motor symptoms but more severe autonomic dysfunction, despite the preservation of dopaminergic innervation.

In summary, the PD-SWEDD group showed several different clinical features compared with the HC group and the PD group. There were no significant differences of MDS-UPDRS III scores after 2 years of follow-up between the MDS-UPDRS III matched PD and SWEDD group, despite the decreased SBR values of the PD group. This may suggest that the PD-SWEDD group is not likely to be early PD. Also, there were significant differences of the putaminal asymmetry indices between the PD group, and the HC and Other-SWEDD groups. It has been suggested that some patients with PD could be misclassified as SWEDD, due to the misinterpretation of presynaptic dopamine images [1]. We suggest that dopaminergic denervation may not be the main pathophysiology for SWEDD, and other major pathophysiology, i.e., the serotonergic system, may be the preferential cause [22, 23]. This would require a long-term experimental study with the aid of other functional imaging modalities.

There are some limitations in our study. The diagnosis of PD was based on the UK PD Brain Bank criteria, [24]. which was established on the evaluation of clinical symptoms and signs of the patient. Currently, there are no specific tests available for the diagnosis, and pathological confirmation is not practical. Therefore, we have evaluated the SBR values of I-123 FP-CIT SPECT, which may be considered an objective value with low interobserver variability. Second, the I-123 FP-CIT SPECT images were acquired from multiple institutions. This could cause variations in the image quality. Quality control was performed by the PPMI core image lab, by maintaining a standard acquisition procedure with assurance guidance. Third, the follow-up period of 2 years may be insufficient to draw definite conclusions, considering the fact that PD progress throughout one’s lifetime. Fourth, though we have performed case-matching analysis between the PD-SWEDD and PD group, the small number of subjects of the PD-SWEDD group may have decreased the statistical power. Lastly, comparison of multiple variables between groups may cause false positive results, which require careful interpretation.

The continuing debate on SWEDD is due to the difficulty surrounding the clinical diagnosis, as well as the uncertainty about whether negative presynaptic dopamine imaging could exclude PD. Here, we focused on the evaluation of SBRs and its related values, as they are the most objective clinical indices for dopaminergic denervation. Our study indicates that a similar degree of symptom duration and motor symptoms between PD and SWEDD contributes to the ambiguity of clinical diagnosis. However, the SBR values, asymmetry indices, and the degree of autonomic dysfunctions indicate that SWEDD has a different nature with PD. We conclude that SWEDD is not early PD, and is a different disease entity. Although diagnostic confirmation is troublesome without neurobiopsy, normal presynaptic dopaminergic image findings could be a reliable method in excluding the diagnosis of PD.
